# Inhibition of the *in vitro* Activities of α-Amylase and Pancreatic Lipase by Aqueous Extracts of *Amaranthus viridis, Solanum macrocarpon* and *Telfairia occidentalis* Leaves

**DOI:** 10.3389/fnut.2021.772903

**Published:** 2021-11-08

**Authors:** Olayinka A. Oluwagunwa, Adeola M. Alashi, Rotimi E. Aluko

**Affiliations:** ^1^Department of Food and Human Nutritional Sciences, University of Manitoba, Winnipeg, MB, Canada; ^2^The Richardson Center for Functional Foods and Nutraceuticals, University of Manitoba, Winnipeg, MB, Canada

**Keywords:** leaf extracts, polyphenolic compounds, α-amylase, pancreatic lipase, enzyme inhibition, fluorescence intensity, circular dichroism

## Abstract

Inhibition of digestive enzymes such as α-amylase and pancreatic lipase (PL) is a promising therapeutic strategy for the treatment and management of chronic health conditions such as diabetes and obesity. Therefore, the aim of this work was to determine the enzyme inhibitory activity of polyphenol-rich aqueous extracts of *Amaranthus viridis* (AV), *Solanum macrocarpon* (SM) and *Telfairia occidentalis* (TO) leaves, which were harvested from plants produced using multiple urea fertilizer doses (0–80 kg N/ha). Fertilizer application was applied at two time points (at planting or 2 weeks after seedling emergence). Leaf extracts were obtained using aqueous extraction (1:20, leaves:water) for 4 h at 60°C followed by centrifugation and freeze-drying of the supernatant. Results showed that the extracts inhibited α-amylase, and pancreatic lipase dose-dependently with TO extracts having significantly (*p* < 0.05) higher inhibitory activities for both enzymes. Fluorescence intensity and circular dichroism spectra in the presence and absence of leaf extracts indicate significant changes to the enzyme protein secondary and tertiary conformations. We conclude that the leaf extracts, especially from TO are potential agents for reducing calorie intake as a preventive or treatment tool against chronic diseases such as diabetes and obesity.

## Introduction

Polyphenol-rich plant foods have been reported to induce insulin-like effects and can act as good inhibitors of enzymes such as α-amylase and pancreatic lipase associated with type 2 diabetes, obesity and lipid peroxidation (1). In order to control the function of these enzymes, larger plants, animals and microorganisms have been found to produce large number of different enzyme protein inhibitors of these enzymes. These enzyme inhibitors block the enzyme's active center thus preventing the rate at which polysaccharides are digested (2–4). Different *in vitro* and *in vivo* studies have shown that dietary phenolic compounds have many beneficial properties in maintaining human health (5). Studies have found that phenolic compounds are good inhibitors of α-amylase, and pancreatic lipase (6–9). The ability of plant-derived products such as from oats (10), berry (11) and tea (12) to inhibit these enzymes are associated with the phenolic content and other flavonoid components (13). One of the major ways of controlling diabetes is by inhibiting a carbohydrate-hydrolyzing enzyme such as α-amylase, which reduces the amount of glucose available for absorption into the body from the small intestine (14, 15). α-amylase cleaves α-1,4 glycosidic bonds to convert complex dietary carbohydrates like starch into oligosaccharides and disaccharides, which are further broken down into absorbable monosaccharides such as glucose and fructose by glucosidases (16, 17). Another enzyme of utmost importance in diabetes is pancreatic lipase (PL), which digests lipids, mainly dietary triacyl-glycerides, which are broken down into monoglycerides and free fatty acids that can be readily absorbed into the blood circulatory system (18, 19). PL inhibition reduces the absorption of fat in the small intestine thus contributing to reduced calorie intake and prevention of excessive body weight gain (20). Therefore, inhibition of α-amylase and PL activities is a known strategy to prevent the breakdown of dietary polysaccharides and fats, which leads to reductions in the absorption of simple sugars and lipids (21, 22). With the global increase in the occurrence of diabetes and obesity, inhibition of these enzymes is of utmost importance in disease management (23).

Fluorescence emission spectroscopy is a useful tool to measure minute structural changes in protein structure, especially upon binding to small molecule ligands such as enzyme inhibitors. This is because aromatic amino acids like Trp, Tyr, and Phe can emit fluorescence spectra with maximum values at 350, 303, and 280 nm, respectively, when they are excited in the UV region (24). However, the emission wavelengths reflect conformational changes in the protein, which are dependent on exposure of the aromatic amino acids to the hydrophilic environment (25). For example, decreases in fluorescence intensity are indications of protein unfolding and increased exposure of aromatic amino acids to a more polar environment while increases suggest shift to non-polar environments (24, 26). Circular dichroism (CD) measures the secondary and tertiary conformations of proteins, which can be used to evaluate relationships of enzyme protein structure to catalytic activity especially in the presence of inhibitors. The CD signal of each protein depends on the number and proximity of the aromatic amino acid residues to each other, degree of H-bonding and presence of disulfide bonds (27).

Foods are well known sources of enzyme inhibitory compounds, but their levels could be dependent on agronomic practices, which are critical for defining crop productivity, nutrient composition, and food availability. Therefore, in order to ensure food supply and increase crop productivity, the positive effect of mineral fertilizers cannot be overemphasized (28, 29). The use of fertilizer micro-dosing gives promising results in terms of crop productivity and farmers' income when compared to the traditional fertilizer broadcasting method. Fertilizer micro-dosing is a method of fertilizer application in small quantities at an optimal depth and distance around the target crop such as leafy vegetables (29, 30) and maize (31) either during the time of planting or some weeks after planting. Water is a universal solvent with numerous advantages as a green extraction solvent because it is cheap, non-flammable, non-toxic, environmentally friendly, and prevent pollution when compared to organic solvents (32). Moreover, the use of water enhances solubility of the extracted polyphenolic compounds within the mainly aqueous-based *in vitro* assay reagents and also in the aqueous gastrointestinal tract when ingested, which ensures bioavailability. Although the organic leaf extract of fluted pumpkin (33), and eggplant (34) have been reported to inhibit α-amylase, there is paucity of information in literature on the inhibition of α-amylase and PL by aqueous extracts of these plants as well as the consequence of phenolic interactions on enzyme structural conformation. Therefore, the aim of this work was to determine the effect of fertilizer micro-dosing on the *in vitro* inhibitory activities of aqueous extracts of *Amaranthus viridis* (AV), *Telfairia occidentalis* (TO) and *Solanum marcrocarpon* (SM) leaves against α-amylase, and PL. The effects of these leaf extracts on the structural conformations of α-amylase and PL were also measured using circular dichroism (CD) and intrinsic fluorescence to determine possible means by which the phenolic compounds attenuate catalysis rate. AV, SM, and TO were chosen for this study because they are one of the most under-utilized indigenous culinary herbs in Nigeria, popularly grown among local farmers. They are commonly consumed by indigenous people as blood boosters, to treat infertility, as anti-inflammatory, antidiabetic and antiviral agents. Moreover, AV, SM, and TO produce a range of polyphenolic compounds including caffeic acid, rutin, and myricetin (30, 35, 36).

## Materials and Methods

### Materials and Chemicals

Porcine pancreas PL (26.9 units/mg protein) and α-amylase Type VI-B (25 units/mg solid) were purchased from Alfa Aesar (Tewksbury, MA, USA) and Sigma Aldrich (St. Louis, MO, USA), respectively, while other analytical grade reagents were from Fisher Scientific (Oakville, ON, Canada). The plants (AV, TO, and SM) were produced at the Micro-Veg Project experimental farm, located at the Obafemi Awolowo University, Ile-Ife, Osun State, Nigeria. The plants were grown using mineral fertilizer application according to the fertilizer micro-dosing technology (~0.1–0.5 cm deep) with a randomized complete block design and five nitrogen (urea) fertilizer doses, which were replicated four times as follows: 0, 20, 40, 60, and 80 kg of urea/ha. Cow manure was used as a base organic fertilizer at 5 t/ha on experimental plots (2 m × 3 m) that received urea at 20, 40, and 60 kg N/ha. In contrast, the plots that received 80 kg N/ha urea did not contain organic fertilizer while the 0 kg N/ha plots received only the organic fertilizer but not urea. The urea fertilizer was applied to each plot during planting (T1) or 2 weeks after emergence of seedlings (T2) to obtain the following samples: 0-T1, 0-T2, 20-T1, 20-T2, 40-T1, 40-T2, 60-T1, 60-T2, 80-T1, and 80-T2. Twenty-five days after seedlings emerged, the leaves were harvested, rinsed in potable water, destalked, and dried using an air cabinet at 60°C for 8 h. The dried leaves were milled into fine powder using a Marlex Excella dry mill (Marlex Appliances PVT, Daman, India) followed by storage at −20°C.

### Preparation of Aqueous Extracts Containing Free Polyphenols

Free water-soluble polyphenolic compounds were extracted using the method of Olarewaju et al. (30). Dried leaf powders were mixed with 20 volumes of distilled water at 60°C for 2 h under constant stirring. After cooling to 25°C, the mixture was centrifuged at 10,000x g for 30 min and supernatant passed through a cheese cloth. The residue was re-extracted and centrifuged using same conditions to obtain a second supernatant. The two supernatants were pooled together, concentrated using a vacuum rotatory evaporator at 60°C, freeze-dried and the extract powder stored at −20°C. We have previously reported that the dried leaf extracts are composed mainly of polyphenols with total polyphenolic content values in the range of 460–611 mg gallic acid equivalent/g where rutin, myricetin, and caffeic acid were detected as the major compounds (30).

### α-Amylase Inhibition Assay

The α-amylase inhibitory activity of leaf extracts was determined using the method described by Karakaya et al. (37) with slight modifications. The enzyme substrate was prepared by bringing to boil 100 mL of distilled water in a 250 mL beaker on a hot plate and then added to a smooth paste of potato starch followed by stirring until it is dissolved. The starch solution was then allowed to cool down to room temperature before it is used for the enzyme assay. The dried leaf extracts were dissolved in 0.02 M sodium phosphate buffer containing 0.006 M NaCl, pH 6.9. A 100 μL aliquot of each sample (assay concentrations of 1.1–2.3 mg/mL) and 100 μL of α-amylase enzyme solution (1 mg/mL) were added to test tubes and allowed to incubate at 37°C for 10 min. A sample blank was prepared with the enzyme omitted. After incubation, 100 μL of 1% (w/v) starch solution was added to test tubes and the reaction mixture incubated at 37°C for 10 min. The reaction was terminated by adding 200 μL of 3,5-dinitro-salicylic acid (DNS) color reagent (96 mM DNSA, 2 M sodium potassium tartrate tetrahydrate and 2 M NaOH) followed by incubation in a boiling water bath at 100°C for 5 min. The reaction mixture was allowed to cool to room temperature, after which 3 mL of MilliQ water was added. A 200 μL aliquot of the reaction mixture was then transferred to a 96-well microplate and the absorbance read at 540 nm using a Synergy™ H4 microplate reader (Biotek™, Vermont, USA) set at 37°C. Acarbose (α-amylase inhibitory drug) at 10 μg/mL was used as the positive control. Percentage inhibitions of all samples were calculated using the equation:


$$\begin{array}{l} \text{Inhibition}\left(\%\right)=\left[\text{Ac}-\left(\text{As}-\text{Asb}\right)/\text{Ac}\right]\times 100 \end{array}$$

Ac = Absorbance of the negative control (uninhibited reaction), As = Absorbance of the sample (inhibited reaction), and Asb = Absorbance of the sample blank (enzyme omitted).

### Pancreatic Lipase Inhibition

PL inhibitory activity of the extracts was determined according to protocols described in previous methods (38, 39) with slight modifications. PL activity was measured using the release of 4-methylumbelliferone (4-MU) from the substrate, which is 4-methylumbelliferyl oleate (4-MU oleate). A 25 μL aliquot of samples (assay activity of 0.5–2.5 mg/mL) dissolved in Tris-buffer (13 mM Tris-HCl, 150 mM NaCl and 1.3 mM CaCl_2_, pH = 8) and 225 μL of a 0.5 mM 4-MU oleate solution were mixed in a 96-well microplate and incubated for 15 min at 37°C. An enzyme blank was prepared with the substrate omitted. After incubation, 25 μL of PL solution (assay concentration = 3.125 U/mL) was added to start the enzyme reaction and then incubated at 37°C for 1 h. After incubation, the amount of 4-methylumbelliferone released by the lipase was measured with a fluorimeter at an excitation wavelength of 340 nm and emission wavelength of 450 nm. Orlistat (PL inhibitory drug at 0.25 mg/mL) served as a positive control and was analyzed using same protocol. The PL inhibitory activity (%) was calculated using the equation:


$$\begin{array}{l} \text{Inhibition}\left(\%\right)=\left[\left(\text{Ac}-\text{Aeb}\right)-\text{As}\right]/\left(\text{Ac}-\text{Aeb}\right)\times 100 \end{array}$$

Ac = Absorbance of the negative control (uninhibited reaction), As = Absorbance of the sample (inhibited reaction) and Aeb = Absorbance of the enzyme blank (substrate omitted). The concentration of extract that reduced enzyme activity by 50% (IC_50_) was obtained by non-linear regression analysis of a plot of PL inhibition (%) vs. the sample concentrations using GraphPad Prism version 9.0 (GraphPad Software, San Diego, CA, USA).

### Intrinsic Fluorescence Emission

The method described by Li and Aluko (40) was used to record intrinsic fluorescence spectra on the Jasco FP-6300 spectrofluorometer (JASCO, Tokyo, Japan) at 25°C with a 1 cm path length cuvette. Sample (leaf extracts) stock solutions (10 mg/mL) and enzymes (PL or α-amylase) were prepared in 13 mM Tris-HCl buffer containing 150 mM NaCl and 1.3 mM CaCl_2_, pH 8 for PL or 20 mM sodium phosphate, containing 6 mM NaCl, pH 6.9 for amylase. The enzyme and sample solutions were then mixed to obtain assay concentrations of 1 mg/mL and 6.25–50 μg/mL, respectively, which were then used for fluorescence emission measurement. The fluorescence spectra were recorded at an excitation wavelength of 275 nm and emission wavelength range of 280–450 nm. Buffer emission spectrum was subtracted from those of the respective samples to obtain reported spectrum of each enzyme/extract mixture.

### Measurement of Circular Dichroism Spectra

The CD spectra of enzyme/leaf extract mixtures (α-amylase and PL) complexes were measured at 25°C in a J-810 spectropolarimeter (JASCO, Tokyo, Japan) using the spectral range of 190–240 nm (far-UV) for secondary structure determinations and 250–320 nm (near-UV) for tertiary structure (41). Stock solutions of extract and that of the enzymes (PL and α- amylase) were prepared as described above for intrinsic fluorescence. The extract and enzyme solutions were mixed to give assay concentrations of 1–3 mg/mL and 1 mg/mL, respectively. The far-UV and near-UV spectra were acquired using 0.05 and 0.1 cm cuvette path lengths, respectively. The reported enzyme spectra were obtained after subtraction of the respective buffer spectrum.

### Statistical Analysis

Triplicate determinations were used to obtain mean values and standard deviations. For statistical analysis, one-way analysis of variance (ANOVA) was carried out. Significant differences (*p* < 0.05) between mean values were determined using the Duncan's multiple-range test. Statistical analyses were performed with the IBM SPSS Statistical package (version 24).

## Results and Discussion

### α-Amylase Inhibition

**Figure 1:**
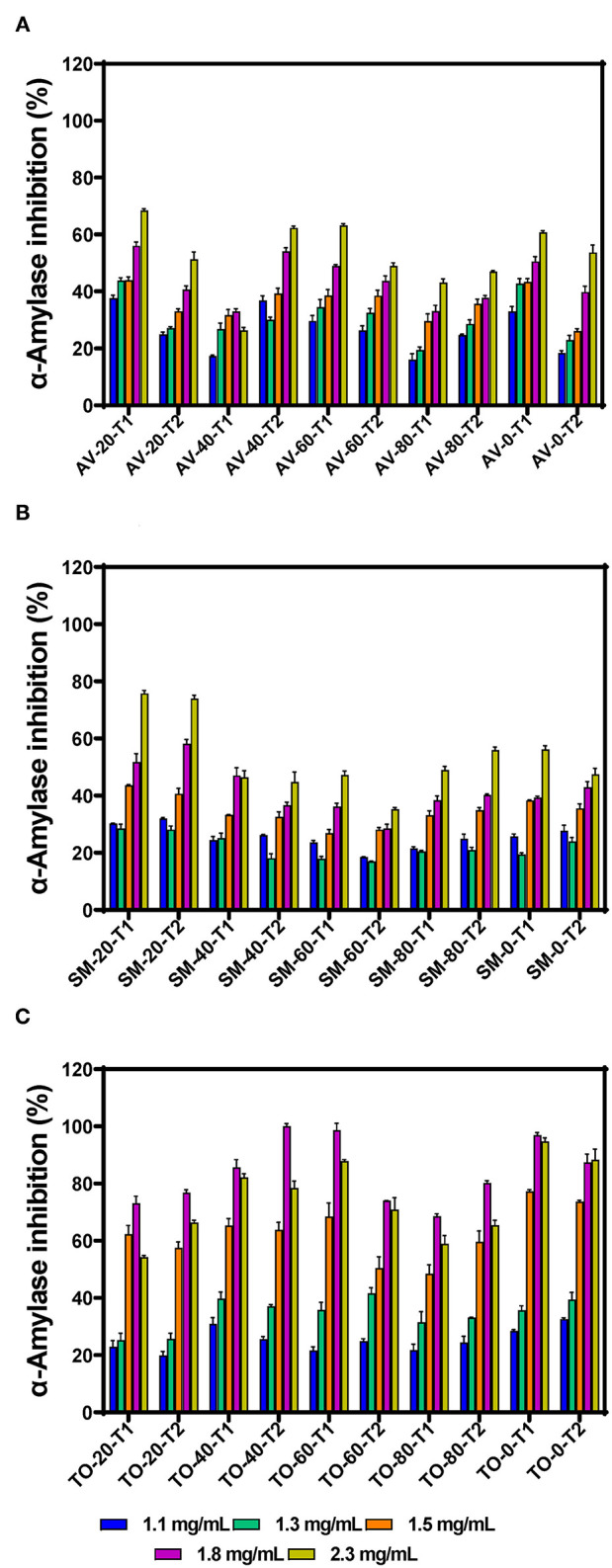
α-amylase inhibition by aqueous extracts from the dried leaves of **(A)** *Amaranthus viridis* (AV), **(B)** *Solanum macrocarpon* (SM) and **(C)** *Telfairia occidentalis* (TO). Plants were produced with different urea fertilizer doses (0, 20, 40, 60, and 80 kg N/ha), which were applied at (T1) or after (T2) planting. Each bar is the mean of 3 determinations while the error bar represents standard deviation.

α-amylase is one of the main enzymes involved in the breakdown of dietary starch, giving rise to oligosaccharides that can be further broken down to absorbable monosaccharides in the brush border of the intestine. Inhibition of this enzyme is therefore considered an active strategy for managing diabetes. The inhibitory activity of the polyphenolic extracts increased with increasing concentration, indicating a dose-dependent effect (Figures 1A–C). This corresponds with the report of Sachan et al. (4), which also reported dose-dependent inhibitory activities for the organic extracts of the medicinal plants *Pluchea lanceolata, Alhagi pseudalhagi*, and *Caesalpinia bonduc*. Among the three vegetable extracts tested, TO-40-T2 (100.00% at 1.8 mg/mL) had the highest inhibition followed by SM-20-T1 (75.74 %) and AV-20-T1 (68.45 %) at 2.3 mg/mL, respectively. However, there was no direct relationship between fertilizer dose and inhibition of α-amylase activity. The inhibitory activity of the extracts was lower than that of the standard acarbose (86.64% at 10 μg/mL). When comparing the three vegetable extracts, it was observed that all the TO extracts showed highest inhibitory activity at 1.8 mg/mL assay concentration after which the activity decreased. This indicate that at concentrations >1.8 mg/mL, there may have been antagonistic interactions between the extract constituents, which led to reduced interactions with the enzyme. This antagonistic effect could be due to increased polyphenol-polyphenol interactions or binding of a compound occupies a space on the enzyme surface or active site, which prevents attachment of other compounds. The results obtained for SM extracts also show weaker inhibitory effects when compared to *Solanum melongena* (40.11%) and *Solanum macrocarpon* (42.66%) at 80 μg/mL as previously reported (34). Differences in activities may be due to variations in cultivar and agronomic practices as well as the extraction media (aqueous vs. organic), which could affect the type and ratio of polyphenolic compounds present in the extracts. Our previous work has showed that the main polyphenolic compounds in the AV, SM, and TO vegetable leaf extracts are caffeic acid, rutin, and myricetin (35, 36). However, the work of Nwanna (34) with SM extracts did not indicate the dominant polyphenols.

The effects of fertilizer dose, time of fertilizer application and vegetable variety differences on the inhibitory activity of the extracts were analyzed using three-way ANOVA (Table 1). The results show that there are significant differences (*p* < 0.05) among the fertilizer dose treatments. The leaf extract from urea fertilizer dose of 80 kg N/ha (53.99%) had the lowest α-amylase inhibition while that of the control treatment (0 kg N/ha—with only organic fertilizer) had the highest inhibition (66.85%). The rate of α-amylase inhibition decreased as the rate of urea fertilizer dose increased indicating a negative effect, which could not be explained by the differences in content of polyphenolic compounds that we previously reported (35, 36). Nitrogen is an important nutrient required for plant growth and metabolism of cellular compounds such as proteins, nucleic acids, ATP, chlorophyll, pigments, and for production of secondary metabolites (42). A study conducted using green and red lettuce indicate that low nitrogen availability increased the concentration of phenolic compounds (43) while in basil leaves (44–46), *Sesamum indicum* (47) and *Hypericum pruinatum* (48), higher nitrogen availability led to a decrease in rosmarinic acid, the main phenolic compounds, which may explain the results obtained in this study. The reduction in α-amylase inhibition could also be explained by the study done by Olarewaju et al. (30), which observed that increase in fertilizer dose led to a decrease in polyphenol content of leaf extracts of AV, SM, and TO. Therefore, the results suggest that the synthesis of polyphenolic compounds is not necessarily supported by higher concentration of nitrogen fertilizer. Thus, precise or optimized targeted use of nitrogen fertilizer could be an effective strategy for enhancing bioactive properties of plants. The time of urea fertilizer application also significantly (*p* < 0.05) affected the rate of α-amylase inhibition by the leaf extracts because application at the time of planting led to significantly (*p* < 0.05) higher (61.01%) value than when applied two weeks after planting (59.42%). Among the three vegetable extracts, TO leaf extracts had significantly (*p* < 0.05) higher inhibition of α-amylase activity when compared to SM and AV extracts. The stronger inhibitory effect of TO extracts may be due to the higher contents of caffeic acid as previously reported (35, 36). The results are consistent with findings that plants contain some chemical substances which are potential inhibitors of α-amylase and due to this they can be used as therapeutic agents or as functional foods in the management of diseases associated with carbohydrate uptake (49).

**Table 1:** Results from 3-way ANOVA and Duncan's test of the effects of vegetable variety, (VV); fertilizer dose, (FD); fertilizer application time, (FAT): T1 at planting, T2 after planting; Telfairia occidentalis, TO; Solanum marcrocarpon, SM; Amaranthus viridis, AV; (0T-80T as fertilizer dosing treatments) on α-amylase inhibitory activity of vegetable leaf aqueous extracts.

**Parameter**	**Mean intensity for VV**			**Mean intensity for FD**					**Mean intensity for FAT**	
	**SM**	**AV**	**TO**	**0T**	**20T**	**40T**	**60T**	**80T**	**T1**	**T2**
**Inhibition of α-amylase activity (%)^1^**	53.19^a^ (0.360)	52.59^a^ (0.360)	74.92^b^ (0.360)	66.85^e^ (0.464)	64.99^d^ (0.464)	56.44^b^ (0.464)	58.91^c^ (0.464)	53.99^a^ (0.464)	61.06^a^ (0.294)	59.42^b^ (0.294)

a−e *Mean intensity values (followed in brackets by the standard error of the mean) within the same variable “vegetable variety,” “fertilizer dose,” and “fertilizer application time” with the same letter within the same row (parameter) are not significantly different (p < 0.05)*.

1 *2.3 mg/mL extract concentration*.

### Pancreatic Lipase Inhibition

**Figure 2:**
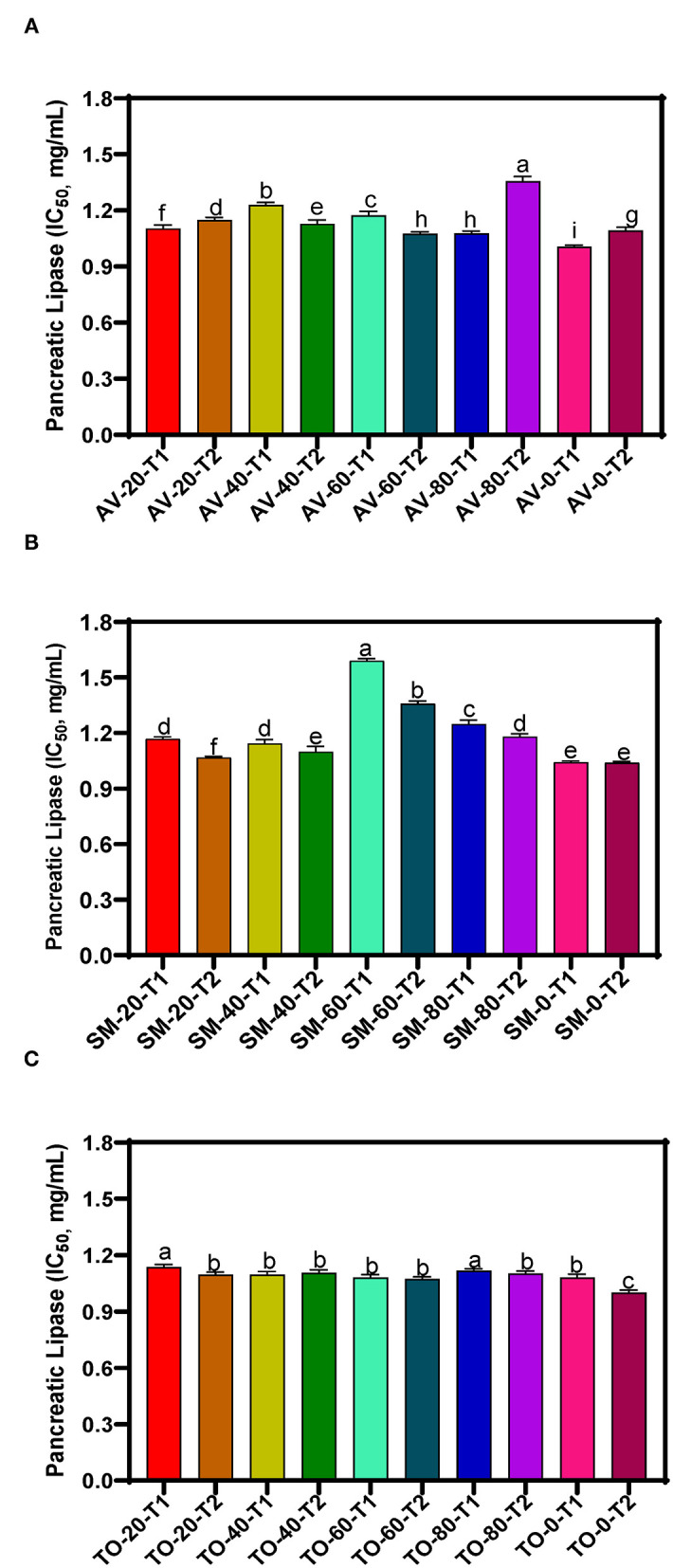
Pancreatic lipase inhibition of aqueous extracts from the dried leaves of **(A)** *Amaranthus viridis* leaves (AV), **(B)** *Solanum macrocarpon* (SM) and **(C)** *Telfairia occidentalis* (TO). Plants were produced with different urea fertilizer doses (0, 20, 40, 60, and 80 kg N/ha), which were applied at (T1) or after (T2) planting. Each bar is the mean of 3 determinations while the error bar represents standard deviation. Bars with different letters (a–g) have mean values that are significantly (*p* < 0.05) different.

The IC_50_ values of samples, which reflect the concentration of the extracts at which 50% of enzyme activity is inhibited (compared to the uninhibited reaction) are presented in Figures 2A–C. Throughout the investigation, the aqueous extracts of TO show the highest PL inhibitory effects but TO-0-T2, AV-0-T1and SM-0-T2 (zero nitrogen fertilizer) had the highest inhibitory activity with IC_50_ values of 1.000, 1.006, and 1.038 mg/mL, respectively, when compared to the other TO, SM and AV samples. The results show very similar values for the TO samples (T1 and T2) while the SM and AV samples had more variations. The results indicate a negative effect of nitrogen fertilizer application on the PL-inhibitory activity of extracts. Therefore, application of urea fertilizer may not be compatible with producing AV, SM, and TO leaves that contain polyphenolic compounds with strong high enzyme-inhibitory potency. The PL inhibition efficacy obtained in this study is lower than those obtained for aqueous extracts of *Vitis vinifera* and *Rhus coriaria* with IC_50_ values of 14.14 and 19.95 μg/mL, respectively (50). The variation in these results may be attributed to the type of method used in determining PL activity and the effect of different bioactive compounds present in the extracts. Other studies have identified natural products for their PL inhibition with profound inhibition effect on fat digestion. Some of these plants include *Ononis natrix* (IC_50_ 167 μg/mL), *Fagonia arabica* (IC_50_ 204.1 μg/mL), *Origanum syriaca* (IC_50_ 234 μg/mL), and *Hypericum triquetrifolium* (IC_50_ 236.2 μg/mL) (51). Teixeira et al. (52) also found that *Passiflora nitida* extract inhibited PL with an IC_50_ value of 21.2 ± 0.8 μg/mL. Although transferring *in vitro* experiment to *in vivo* experiment might not bring out the same outcome but with the high inhibition rate of the leaf extract of AV, SM, and TO, conducting an *in vivo* study using this leaf extracts might pave way for a better understanding of their mechanism of action, possible side effect and their optimal dose. Establishing this will give rise to a more effective and safer strategy in the management or treatment of obesity.

The three-way ANOVA results (Table 2) revealed a significant difference in the inhibitory activity of the extracts of AV, SM, and TO. Extracts with fertilizer dose of 60 kg N/ha had the lowest PL inhibitory activity while the control treatment (0 kg N/ha) had the highest inhibitory effect among all the samples. This shows that extracts from the control treatment (no nitrogen fertilizer) are better inhibitors of PL when compared with extracts that contain both organic and urea fertilizers (20, 40, and 60 kg N/ha). Among the three leaf extracts studied, TO have the best inhibitory effect when compared with the other two vegetables (SM and AV). The time of fertilizer application significantly affected the rate at which the extracts inhibited PL activity. Leaf extracts from plants that received fertilizer treatment at the time of planting (T1) were better inhibitors of PL than those from plants fertilized two weeks after planting (T2). The reason is not clear but could be that early fertilizer application ensured faster synthesis and accumulation of inhibitory polyphenolic compounds than the late treatment.

**Table 2:** Results from 3-way ANOVA and Duncan's test of the effects of vegetable variety, (VV); fertilizer dose, (FD); fertilizer application time, (FAT): T1 at planting, T2 after planting; *Telfairia occidentalis*, (TO); *Solanum marcrocarpon*, (SM); *Amaranthus viridis*, (AV); (0T-80T as fertilizer dosing treatments) on pancreatic lipase inhibitory activity of vegetable extracts.

**Parameter**	**Mean intensity for VV**			**Mean intensity for FD**					**Mean intensity for FAT**	
	**SM**	**AV**	**TO**	**0T**	**20T**	**40T**	**60T**	**80T**	**T1**	**T2**
**Inhibition of pancreatic**	76.125^b^	74.57^a^	77.71^c^	82.14^d^	74.51^b^	76.77^c^	71.28^a^	75.97^b^	76.59^a^	75.92^b^
**lipase activity^1^**	(0.275)	(0.275)	(0.275)	(0.355)	(0.355)	(0.355)	(0.355)	(0.355)	(0.225)	(0.225)

a−d *Mean intensity values (followed in brackets by the standard error of the mean) within the same variable “vegetable variety,” “fertilizer dose,” and “fertilizer application time” with the same letter within the same row (parameter) are not significantly different (p < 0.05)*.

1 *2.5 mg/mL extract concentration*.

### Intrinsic Fluorescence Emission

#### α-Amylase

**Figure 3:**
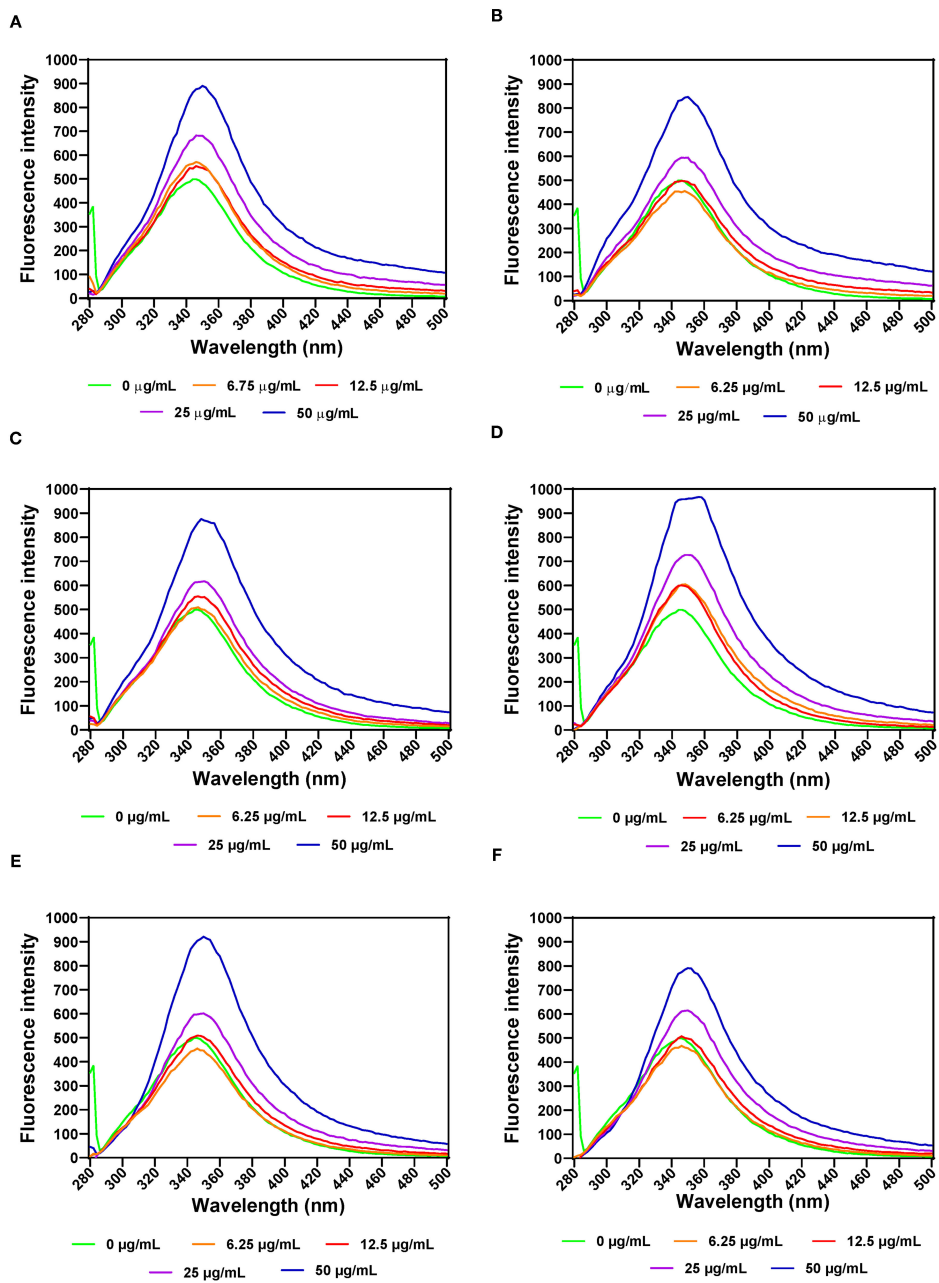
Intrinsic fluorescence intensity of α- amylase in the presence of varied concentrations of leaf extracts from: **(A)** AV-20-T1, **(B)** AV-20-T2, **(C)** SM-20-T1, **(D)** SM-20-T2, **(E)** TO-20-T1, and **(F)** TO-20-T2. Plants were produced with nitrogen fertilizer (20 kg N/ha), which was applied at (T1) or after (T2) planting.

The effects of leaf extracts on α-amylase conformational changes were evaluated by intrinsic fluorescence intensity measurements using samples with same nitrogen fertilizer treatment (20 kg N/ha). Changes in the emission spectra of tryptophan are common in response to protein conformational transitions, subunit association, substrate binding, or denaturation (53). The results show varied outcomes but mostly increases in the fluorescent intensity (FI) at higher concentration of leaf extracts (Figures 3A–F). Decreases in the FI represent the unfolding of the protein structure and exposure of tryptophan residues to a more polar environment while increases suggest shift to non-polar environments (24, 26). The results suggest that at some concentrations, addition of the extracts led to a loose α-amylase protein conformation with greater exposure to the hydrophilic environment, hence FI quenching. However, for all the three leaf varieties at the highest concentration of 50 μg/mL, the α-amylase aromatic amino acids may have interacted with the polyphenolic compounds in the extract, which reduced exposure to the hydrophilic environment, hence increased FI. α-amylase alone had wavelength of maximum FI (λmax) at 344 nm, which was red shifted after addition of the extracts. The red-shift indicates changes to the tryptophan microenvironment with greater exposure to hydrophilic residues upon binding with polyphenols in the extracts (24). At 6.25 and 12.5 μg/mL, a shift from 344 to 346 nm occurred for all the extracts except for AV-20-T2, which instead shifted to 348 nm. However, at 25 and 50 μg/mL, there was increased red shift to 350 nm for all the extracts. This suggests that the tryptophan residues inside the protein molecules were more exposed to the protein surfaces thus making the tryptophan environment more polar (54). Results showing changes in FI and λmax indicate that the α-amylase inhibitory activity of the AV, SM, and TO leaf extracts as shown in Figure 1 is due to ability of the extracts to change the enzyme protein structure from a conformation that facilitated substrate catalysis into those not favorable for optimum enzyme activity. Interaction of polyphenols with enzymes could bring about changes in the emission spectra of the enzymes due to polyphenol binding. The reaction of polyphenol compounds such as epigallocatechin-G, epigallocatechin, epicatechin-G, naringenin, kaempferol-glu, caffeic acid, rosmarinic acid, and p-coumaric acid with bovine serum albumin (BSA) shows that, these polyphenols can quench the intrinsic fluorescence of BSA. Titration of epigallocatechin-G, epigallocatechin, epicatechin-G with BSA showed a higher shift in the emission spectra indicating a more polar environment for tryptophan while the emission spectra of BSA was not significantly affected by other polyphenols (55). This suggest that the result obtained in our study could be attributed to activity of the polyphenol compounds (rutin, caffeic acid, and myricetin) present in the AV, SM, and TO leaf extracts. Therefore, the changes observed in the FI at different concentrations of the AV, SM, and TO leaf extract may be due to the direct quenching or as a result of enzyme conformational changes induced by the reaction of polyphenol compounds with α-amylase protein (55). It is also possible that hydrogen bonding occurred between the polyphenols of the AV, SM, and TO leaf extracts and α-amylase thus altering the microenvironment of the intrinsic chromophore groups of the enzyme (56, 57).

#### Pancreatic Lipase

**Figure 4:**
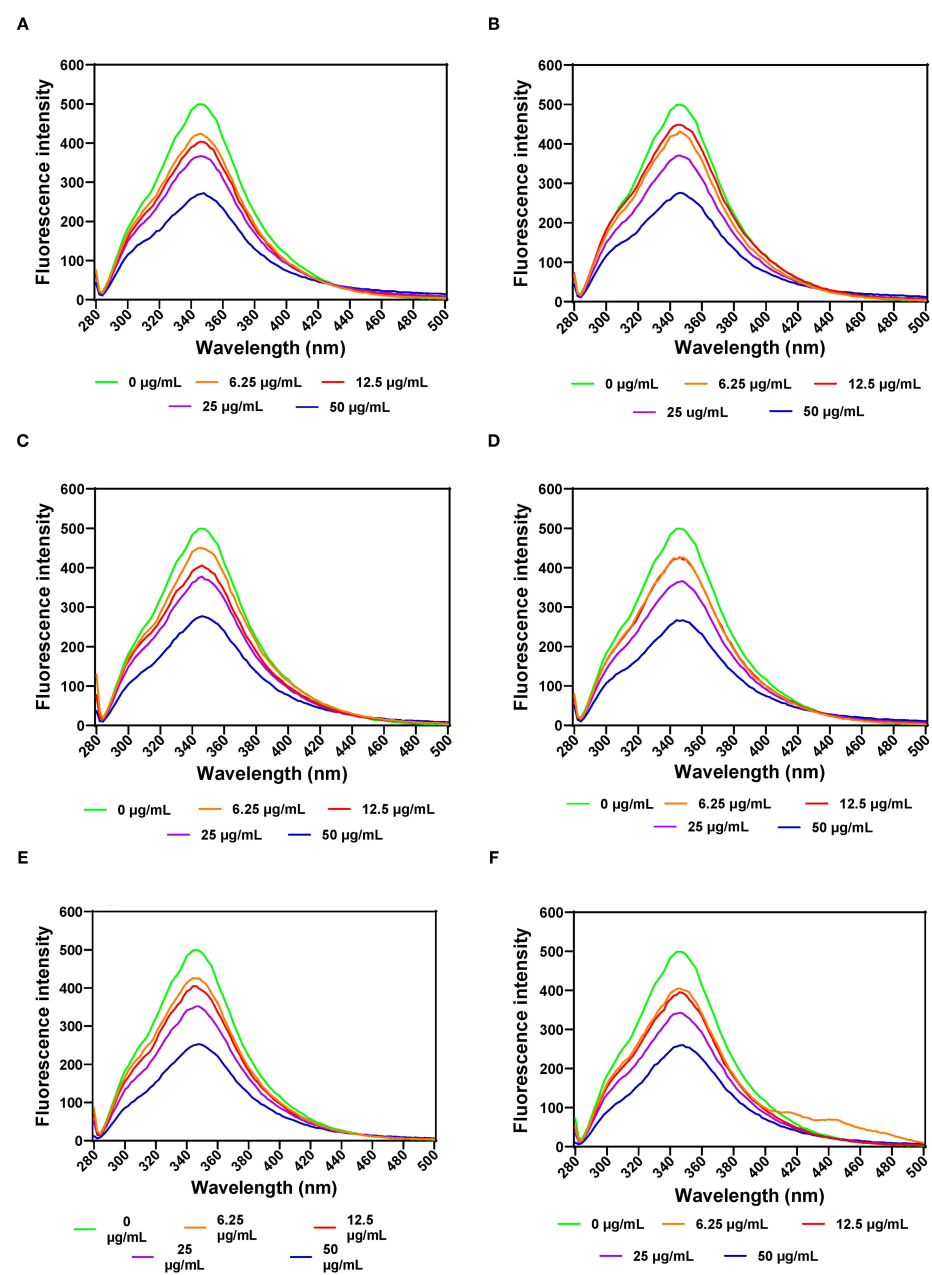
Intrinsic fluorescent intensity of pancreatic lipase in the presence of varied concentrations of leaf extracts from: **(A)** AV-40-T1, **(B)** AV-40-T2, **(C)** SM-40-T1, **(D)** SM-40-T2, **(E)** TO-40-T1, and **(F)** TO-40-T2. Plants were produced with nitrogen fertilizer (40 kg N/ha), which was applied at (T1) or after (T2) planting.

FI was also used to evaluate PL structural changes as a result of addition of various leaf extract concentrations. The results show that increases in the concentration of AV, SM, and TO leaf extracts led to decreased FI, which indicate conformational changes that exposed the aromatic amino acids to a more polar environment (Figures 4A–F). PL exhibited a single fluorescence emission peak at 346 nm, which is due to the presence of tryptophan amino acid residue. Therefore, decreases in FI suggest unfolding of the PL protein molecule, which led to increased tryptophan interactions with the hydrophilic environment. Addition of SM-40-T1 and TO-40-T1 resulted in a slight blue shift of λmax to 344 nm and SM-40-T2 exhibited a red shift to 348 nm at 6.25 μg/mL. A similar result was reported for the interaction between caffeic acid and PL (58). The blue shift obtained upon addition of the AV, SM, and TO leaf extracts indicates changes that moved the tryptophan into a more hydrophobic environment (24, 59). This suggests that there was structural reorganization that led to unfolding of enzyme molecule accompanied by changes in the microenvironment of tryptophan and tyrosine residues in the protein (54, 59). The lower FI values observed for the enzyme interaction with 50 μg/mL of AV, SM, and TO leaf extracts show that the tryptophan residues were most exposed to the polar environment, which represented the loosest conformation when compared to the lower concentrations (58, 60, 61). A previous study reported similar results, which showed that the fluorescence intensity of pancreatic lipase decreased gradually at increased concentrations of quercetin, isoquercetin, and rutin with a slight blue shift from 354 to 351 nm (62). This suggests that flavonoids could expose tryptophan residue to a more hydrophobic environment and lead to attenuated fluorescence intensity of lipase. Gonçalves et al. (63) also reported a decrease in the FI of PL upon interactions with grape seed procyanidins but with no changes to the structure of PL. In another study, a different result was obtained, which reported an increased FI for the interactions of PL with galangin, kaempferol, quercetin, and myricetin (64). The results obtained in this study suggests that the fluorescence of PL was quenched by vegetable extracts as a results of protein structural unfolding, which is consistent with the observed reductions in PL activity.

### Far-UV Circular Dichroism Spectra

#### α-Amylase

**Figure 5:**
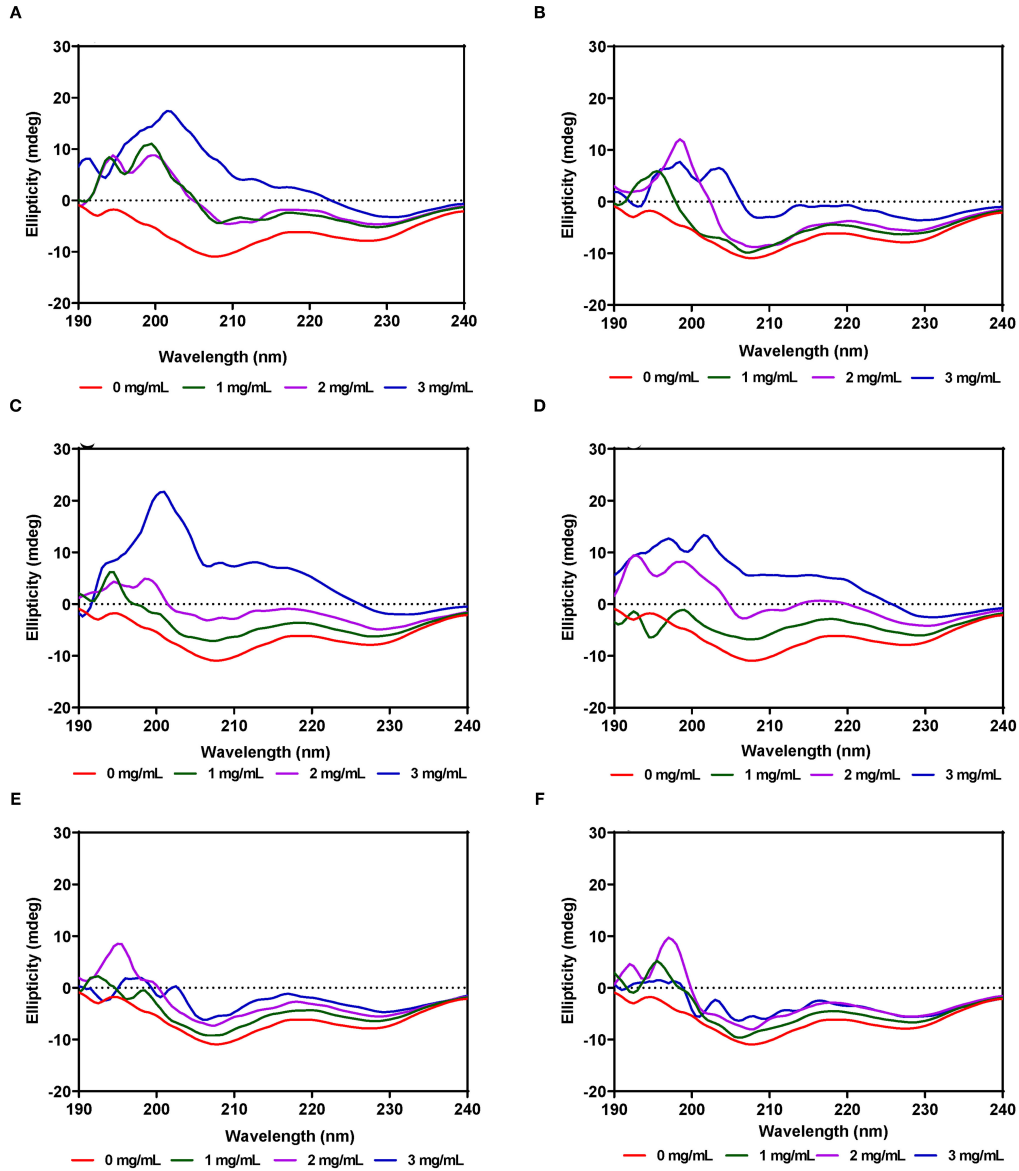
Far-UV CD of α-amylase in the presence of varied concentrations of leaf extracts from: **(A)** AV-20-T1, **(B)** AV-20-T2; **(C)** SM-20-T1, **(D)** SM-20-T2, **(E)** TO-20-T1, and **(F)** TO-20-T2. Plants were produced with nitrogen fertilizer (20 kg N/ha), which was applied at (T1) or after (T2) planting.

The secondary structure of α-amylase in the presence of AV, SM, and TO leaf extracts was investigated using CD measurements between 190 and 240 nm at extract concentrations of 1, 2, and 3 mg/mL. The CD spectrum of α-amylase alone indicate a typical signal of a protein containing both α-helix and β-sheet as evident by the negative peaks at about 206–208 and 229–231 nm, and a positive peak in the 193–200 nm region (Figures 5A–F), which is similar to a previous report (65). The α-amylase spectrum showed that the 206–208 and 229–231 nm peaks were reduced upon addition of the AV, SM, and TO leaf extracts, which suggest conformational changes that led to altered secondary structures. However, the intensity in the 193–200 nm region increased upon addition of the SM-20-T1, TO-20-T1, and TO-20-T2 extracts. The result is similar to data from a previous work which showed increased intensity of 193–200 nm region of α-amylase CD spectra upon addition of a diosgenin from *Dioscorea bulbifera* (65). This shows that interaction of the leaf extracts with enzyme resulted in changes in the secondary structure conformation of α-amylase. As observed for the intrinsic fluorescence data, addition of 50 μg/mL of leaf extracts resulted in the most change in ellipticity, especially for AV and SM samples. In contrast, changes to the α-amylase CD spectra were minimal upon addition of TO extracts.

#### Pancreatic Lipase

**Figure 6:**
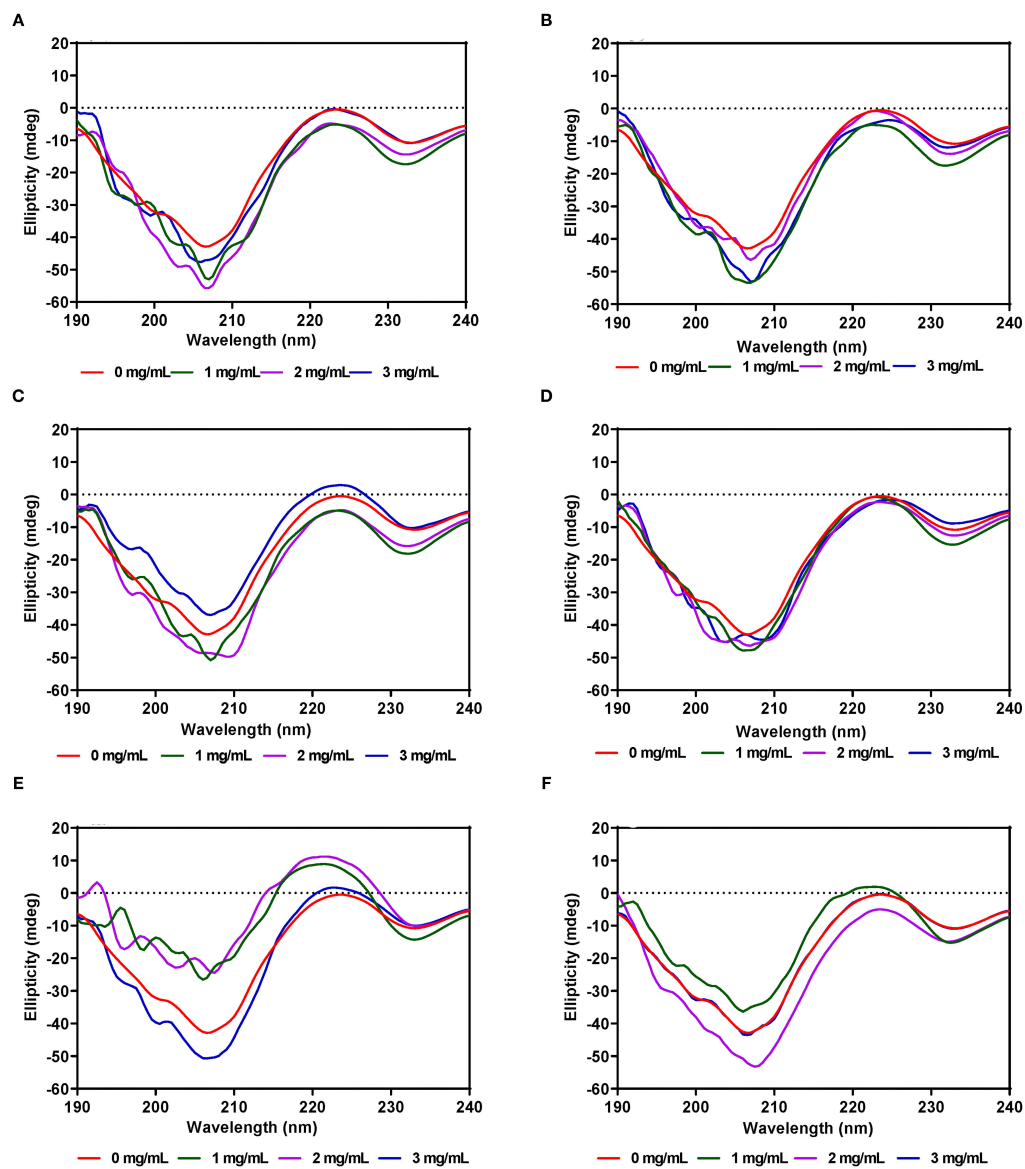
Far-UV CD of pancreatic lipase in the presence of varied concentrations of leaf extracts from: **(A)** AV-40-T1, **(B)** AV-40-T2, **(C)** SM-40-T1, **(D)** SM-40-T2, **(E)** TO-40-T1, and **(F)** TO-40-T2. Plants were produced with nitrogen fertilizer (40 kg/ha), which was applied at (T1) or after (T2) planting.

Unlike α-amylase, secondary structural changes to PL conformation in the presence of the AV, SM, and TO leaf extracts were modest with very minimal changes for AV-40-T1, AV-40-T2, SM-40-T1 and SM-40-T2 (Figures 6A–F). However, addition of the TO-40-T1 and TO-40-T2 extracts led to greater changes in ellipticity at 206–208 and 229–231 nm, which suggest ability to modulate enzyme secondary structure. The ability of TO extracts to produce greater modification of PL secondary structure is consistent with the observed stronger enzyme activity inhibition when compared to the AV and SM extracts. Results obtained in this work are consistent with previous reports that showed changes in α-glucosidase (65), bovine serum albumin (66) and α-lactalbumin (67) proteins secondary structure in the presence of polyphenolic compounds. For example, in the presence of tea epigallocatechin-3-gallate, there was a significant increase in the 190–200 nm peak of α-lactalbumin when compared to the same protein alone.

### Near-UV CD Spectra

#### α-Amylase

**Figure 7:**
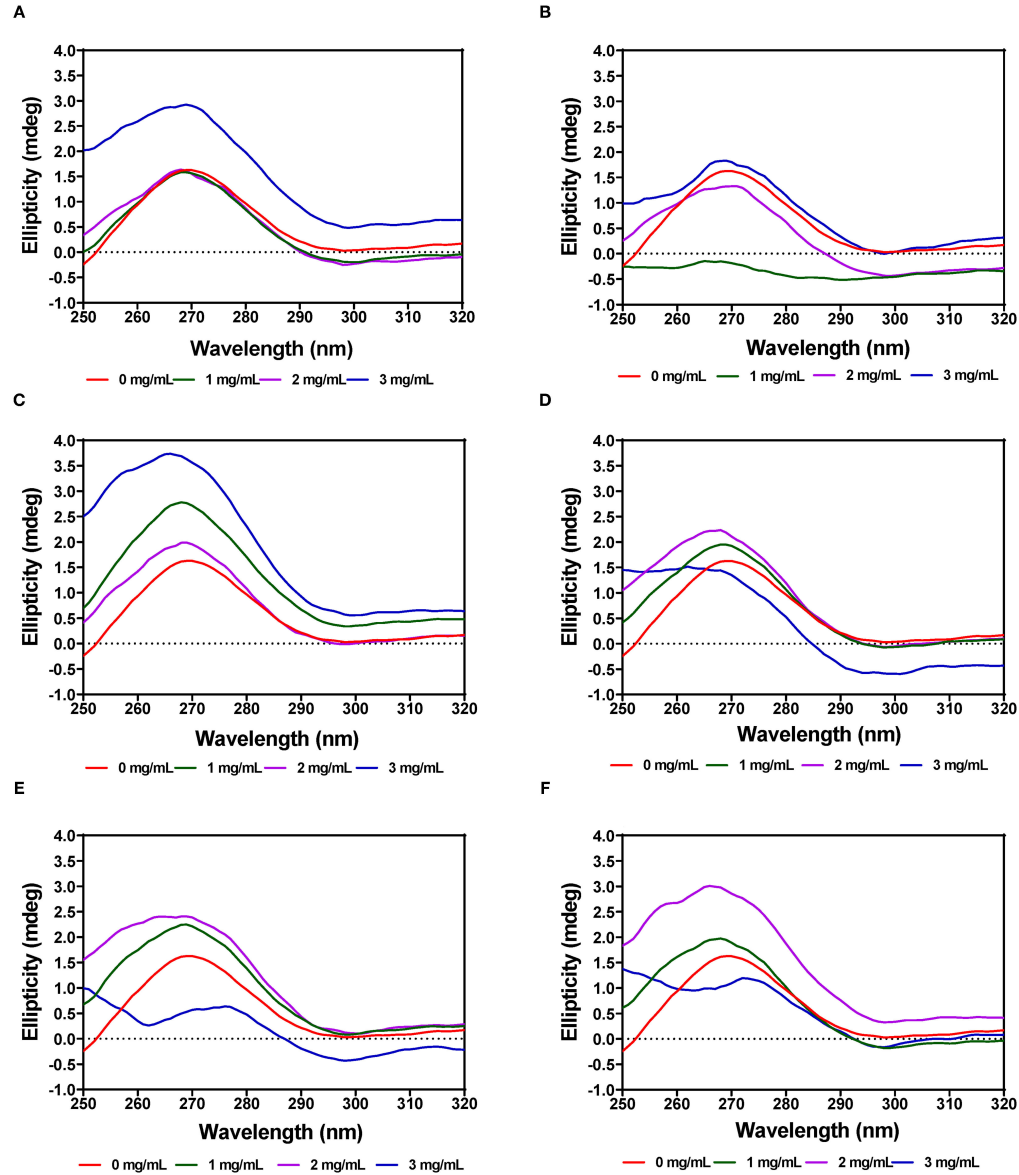
Near-UV CD of α-amylase in the presence of varied concentrations of leaf extracts from: **(A)** AV-20-T1, **(B)** AV-20-T2, **(C)** SM-20-T1, **(D)** SM-20-T2, **(E)** TO-20-T1, and **(F)** TO-20-T2. Plants were produced with nitrogen fertilizer (20 kg N/ha), which was applied at (T1) or after (T2) planting.

The tertiary structure of the α-amylase in the presence of AV, SM, and TO leaf extracts was evaluated using near-UV CD. The CD spectra show that the structural conformation of the enzymes was significantly affected at different extract concentrations of AV, SM, and TO. The spectra for α-amylase showed a prominent positive peak ellipticity at 269–270 nm and weak negative peak ellipticity at 296–298 nm (Figures 7A–F). Addition of 1 and 2 mg/mL AV-20-T1 did not change α-amylase conformation but the 3 mg/mL produced an increased ellipticity peak in the 269–270 nm, which indicate structural changes that moved the aromatic groups away from the hydrophilic surface into more asymmetric environments when compared to the native enzyme. Addition of diogenin was also shown not to change the near-UV spectra of α-amylase (65). In contrast, addition of 1 mg/mL AV-20-T2 led to enzyme conformation changes that exposed the aromatic amino acids into less asymmetric environments as evident in the near-zero ellipticity. Increased concentrations (2–3 mg/mL) of AV-20-T2 produced no substantial changes in α-amylase conformation. The SM-20-T1, SM-20-T2, TO-20-T1, and TO-20-T2 extracts also produced changes in enzyme structure as shown by the increases or decreases in ellipticity peak at 269–270 nm. Addition of varying levels of the AV, SM, and TO leaf extracts led to significant reductions in the 269–270 nm peak, suggesting a concentration-dependent modification of enzyme protein conformation. The observed larger losses of structural rigidity and asymmetric environments of α-amylase in the presence of SM and TO extracts are consistent with the stronger enzyme inhibitory effects when compared to the AV extracts.

#### Pancreatic Lipase

**Figure 8:**
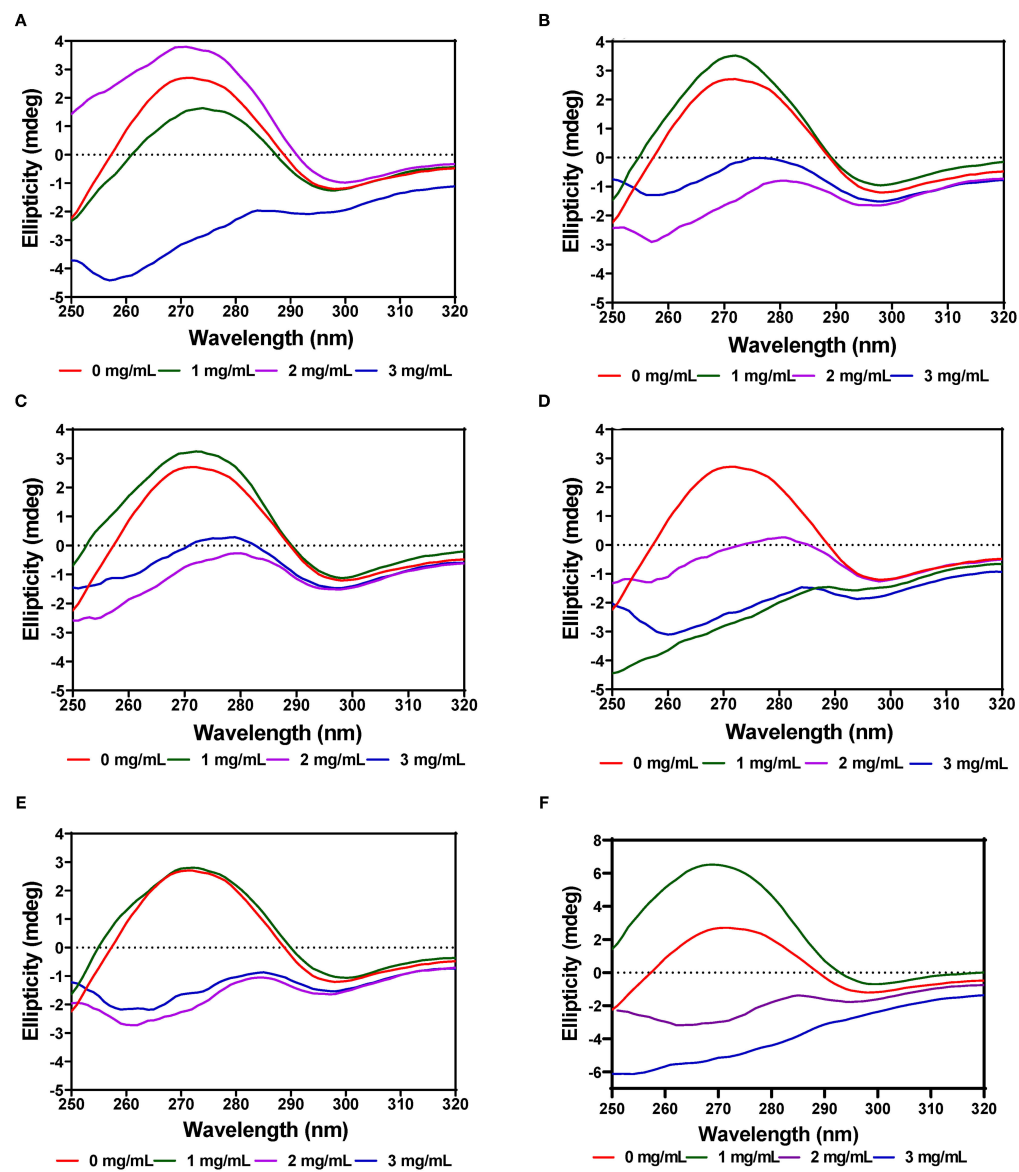
Near-UV CD of pancreatic lipase in the presence of varied concentrations of leaf extracts from: **(A)** AV-40-T1, **(B)** AV-40-T2, **(C)** SM-40-T1, **(D)** SM-40-T2, **(E)** TO-40-T1, and **(F)** TO-40-T2. Plants were produced with nitrogen fertilizer (40 kg/ha), which was applied at (T1) or after (T2) planting.

The near-UV CD spectra for PL showed a positive peak ellipticity at 272–274 nm and negative peak ellipticity at 298–300 nm, which indicate that tyrosine and tryptophan are the main contributors to the observed structure (Figures 8A–F). Addition of AV-40-T1 led to disappearance of the 272–274 nm and 298–300 nm peaks, which indicate relocation of the aromatic amino acid residues to a more hydrophilic or less asymmetric environment. At 2–3 mg/mL, all the extracts (AV, SM, and TO) showed this type of behavior of significant reductions in the two peaks present in the native enzyme, which also suggest significant modifications to PL enzyme structure. The results are consistent with previous studies that have also shown interactions of polyphenols with enzymes can result in conformational changes to the tertiary structure of enzymes (68–71).

## Conclusions

The aqueous extracts of AV, SM, and TO leaves had strong inhibitory activities against α-amylase and PL. Fluorescence intensity analysis coupled with CD indicate that in the presence of leaf extracts, there were changes to enzyme structure that could have been responsible for the observed enzyme activity modulation. Based on fluorescence emission patterns, α-amylase interactions with the extracts indicated shifting of aromatic groups to non-polar environments, which contrasted that of PL where the groups became exposed to hydrophilic environments. Therefore, mechanism of catalysis inhibition by the polyphenolic-rich leaf extracts was enzyme specific, but nitrogen fertilizer application led to decreased inhibitory efficacy of the leaf extracts against α-amylase and PL. Hence the application of urea fertilizer may not be an efficient way of enhancing the enzyme-inhibitory activities of AV, SM, and TO leaf polyphenolic extracts. Overall, the TO extracts had the strongest inhibitory effects, which were reflected in more extensive changes to the secondary and tertiary structures of the enzymes. Inhibitions against these two enzymes by the leaf extracts suggest their potential use as agents that could down regulate blood glucose through reduced intestinal digestion of nutrient polysaccharides and an additional suppression of calorie intake by reducing fat digestion. Therefore, consumption of these leaf extracts, especially TO may interfere with digestive functions that lead to reduced blood glucose and lipids, which could enable body weight control. However, animal and human feeding experiments are required to confirm these *in vitro* data. Moreover, additional studies are required to determine inhibition of glucose transporters 2, because of the potential competition between polyphenolic compounds and glucose for these bioreceptors.

## Data Availability Statement

The original contributions presented in the study are included in the article/supplementary material, further inquiries can be directed to the corresponding author/s.

## Author Contributions

RA: conceptualization, funding acquisition, and project administration. RA, AA, and OO: methodology. RA: resources. OO: writing—original draft preparation. RA and AA: writing—review and editing. OO: formal analysis. RA and AA: supervision. All authors have read and agreed to the submitted version of the manuscript.

## Funding

This research was funded by the International Development Research Center and Global Affairs Canada through the Canadian International Food Security Research Fund, Project 107983 on synergizing indigenous vegetables and fertilizer micro-dosing innovations among West African farmers.

## Conflict of Interest

The authors declare that the research was conducted in the absence of any commercial or financial relationships that could be construed as a potential conflict of interest.

## Publisher's Note

All claims expressed in this article are solely those of the authors and do not necessarily represent those of their affiliated organizations, or those of the publisher, the editors and the reviewers. Any product that may be evaluated in this article, or claim that may be made by its manufacturer, is not guaranteed or endorsed by the publisher.
